# Full Respiration Rate Monitoring Exploiting Doppler Information with Commodity Wi-Fi Devices

**DOI:** 10.3390/s21103505

**Published:** 2021-05-18

**Authors:** Chendan Dou, Hao Huan

**Affiliations:** School of Information and Electronics, Beijing Institute of Technology, Beijing 100081, China; douchendan@bit.edu.cn

**Keywords:** channel state information (CSI), respiration sensing, Doppler shift, Wi-Fi

## Abstract

Respiration rate is an essential indicator of vital signs, which can demonstrate the physiological condition of the human body and provide clues to some diseases. Commercial Wi-Fi devices can provide a non-invasive, cost-effective and long-term respiration rate-monitoring scheme for home scenarios. However, previous studies show that the breathing depth and location may affect the detectability of respiratory signals. In this study, we leverage the variation of the Doppler spectral energy extracted from the channel state information (CSI) collected by Wi-Fi devices to track the chest displacement induced by respiration. First, the random phase is eliminated by phase-fitting method to obtain the complex CSI containing the Doppler shift. Then, the multipath decomposition of CSI is carried out to obtain the channel impulse response, which eliminates the interference phase of the time delay and retains the Doppler shift. The dynamic path units are also separate from the multipath, which overcomes the indoor multipath effect. Finally, we conduct a time–frequency analysis to dynamic units to accumulate Doppler spectral energy. Based on these ideas, we design a complete respiration rate-monitoring system to obtain the respiration rate by using the consistency between the Doppler energy change period and the respiratory cycle. We evaluate our system through extensive experiments in several typical home environments filled with multipath. Experimental results show that the errors of the three scenarios are approximate, the maximum error is less than 0.7 bpm, and the average errors are approximately 0.15 bpm. This result indicates that our scheme can achieve high precision respiration monitoring and has good anti-multipath ability compared with existing methods.

## 1. Introduction

The past few years have witnessed an evolution of sensing technology and the improvement of people’s awareness of their own health; thus, people prefer ubiquitous health monitoring in the daily life to improve the quality of life. Respiration rate, one of the health metrics of vital sign, provides feedback for physiological conditions, such as sleep quality, cardiac arrest and sleep apnoea [1,2]. Respiration rate monitoring is important for early detection of symptoms of chronic respiratory diseases, asthma and chronic obstructive pulmonary disease [3,4,5]. Traditional respiration detection requires specialised medical equipment, such as a breathing monitor with a mask or nasal tube [6,7], which is expensive, invasive and unsuitable for application in a home setting. Some wearable sensors [8,9,10] attached to the waist or chest and pressure sensors embedded in the mattress [11] require an uncomfortable contact with the body, which limit the range of motion and are not suitable for long-term monitoring. The deliberate measurement may also cause people to unconsciously control breathing, affecting the detection effect.

Accordingly, the non-invasive respiration-monitoring scheme based on radio frequency has attracted extensive attention. This scheme uses electromagnetic waves that travel through space to observe breathing movements, and no device is required. Doppler radar [12,13] and ultra-wide band (UWB) radar [14,15] can identify human heart rate and breathing rate. Frequency-modulated continuous wave (FMCW) radar [16] can simultaneously monitor the vital signs of multi-user. These systems allow for more precise measurements; however, they require expensive customised hardware that prevents large-scale and long-term deployment in home settings. In such a scenario, the detection scheme based on Wi-Fi signal provides a wide range of application prospects and great potential because of its economic efficiency, easy deployment and long-term sustainability. Channel state information (CSI) represents the fine-grained channel information generated during the transmission of Wi-Fi signals, which is calculated by a Wi-Fi Network Interface Card (NIC) for channel estimation and equalisation.

Liu et al. [17] used the amplitude of CSI to track breathing and heartbeat during sleep. PhaseBeat [18] and TensorBeat [19] used the CSI phase difference information from the two antennas to detect the vital signs. These studies experimentally confirmed that CSI information can sense respiration, but it did not provide theoretical foundation for the reason. Wang et al. [20] analysed the influence of breathing on Wi-Fi signal propagation on the basis of the theory of Fresnel zone, proposed and verified the theoretical model of breathing sensing and revealed that the detectability of breathing signals is related to breathing depth and location. To solve the problem that respiration could not be effectively detected in the bad places, FullBreathe [21] found that the CSI amplitude and phase are complementary to each other. When the phase change caused by breathing depth is less than π/2, the amplitude and phase of CSI of the two antennas are combined to eliminate the blind spot. However, different depths of breath cause varying phase changes. The phase change caused by normal breathing signal is not always less than π/2. In other cases, the amplitude and phase cannot effectively detect breathing.

To address this key challenge, we present a new respiration-sensing scheme in this study to extract the Doppler features from CSI, which can be used to detect respiratory movement. We first introduced the respiration-sensing principle based on the Fresnel model and found that the CSI amplitude and phase could not always effectively detect breathing signals. Borhani et al. [22] used a radio channel sounder at 5.9 GHz to study the influence of falling and other actions on the indoor channel characteristics, and verified that the Doppler information can be extracted from the time-frequency channel properties. Objects in motion produce Doppler effect, which inspires us to leverage the Doppler shift induced by chest movement during breathing to perceive breathing. Therefore, we establish a respiration-sensing model based on Doppler shift. However, the multipath effect in the indoor environment is severe, and the faint Doppler shift generated by breathing is easily submerged in the static clutter; accordingly, it is difficult to extract it directly from CSI. We propose a Doppler spectral energy extraction method to solve this problem. We first perform multipath decomposition to every CSI by using inverse fast Fourier transform (IFFT) and obtain CIR. Only path units containing dynamic components are retained. Then, the time–frequency analysis of each path unit is carried out to obtain the spectrogram of the Doppler frequency shift. The breathing signal is extracted from the accumulated spectral energy change of Doppler shift at zero frequency according to the periodicity of breathing action.

We design a complete set of respiration rate-monitoring system by using commodity Wi-Fi device on the basis of the analytical result. First, our system detects the presence of breathing in the environment. If a breathing signal is detected, then the amplitude and phase of raw CSI are preprocessed to eliminate noise and phase offset. The clear complex CSI is obtained. Then, the Doppler information generated by chest movement is obtained by multipath decomposition and short time Fourier transform (STFT) of the dynamic path units. Finally, the periodic breathing signal is extracted, and the respiration rate is calculated by peak-to-peak distance method. We implement our system by using a pair of commercial Wi-Fi devices and conduct extensive experiments in three typical home environments (living room, bedroom and balcony) to evaluate its performance. The experimental results demonstrate that the system can achieve high accuracy for respiration rate estimation in different scenarios. The mean estimation errors in three scenarios are 0.16, 0.15 and 0.14 bpm. We also evaluated the impact of different influencing factors on the system.

The main contributions of this study are summarised as follows:

1.  A respiration-sensing model based on the Doppler frequency shift was proposed to solve the problem that the breathing depth and location may affect the detectability of CSI amplitude and phase. The feasibility of extracting Doppler frequency shift for respiration monitoring is theoretically verified.
2.  In view of the phenomenon that Doppler caused by weak chest movement in indoor environment is interfered by multipath effect and cannot be directly extracted, we propose a Doppler spectral energy extraction method to effectively extract breathing signals, including multipath decomposition, dynamic path unit extraction and Doppler shift energy accumulation.
3.  We design a complete respiration rate-monitoring system and conduct extensive experiments to verify and evaluate its performance in actual indoor home environment. The results demonstrate that our system can accurately estimate the respiration rate and achieve developed performance compared with the existing respiration-sensing system.

Section 2 discusses related work. Section 3 provides basic theory of Wi-Fi signal respiration sensing and our model of respiratory signal extraction with Doppler. Section 4 elaborates the design and implementation of our respiration rate-monitoring system. Section 5 demonstrates the experimental results and performance evaluation. Section 6 concludes the paper.

## 2. Related Work

This work aims to measure human respiration. Before that, we briefly review the previous work related to respiration sensing.

### 2.1. Contact-Based Respiration Sensing

In a clinical scenario, respiration is measured by using special equipment. For example, capnometer [6], which is capable of measuring the concentration of CO_2_ in the breathing gas, requires the patient to wear a mask or nasal cannula. The three primary methods to measure breath by wearable sensor are as follows: elastomeric plethysmography, impedance plethysmography and respiration inductive plethysmography [8]. These technologies use sensors attached to the chest and abdomen [9,10] to sense chest and abdominal volume changes or changes in the physical properties of the body surface resulting from expansion and contraction during breathing. Pressure sensors built in the mattress or chair [11,23] can detect the tiny deformations caused by human breathing, thereby extracting respiratory signals. These methods can only detect breathing when the device is in contact with the body. Restricted range of movement in the vicinity of the device does not provide continuous detection, and contact with the body is uncomfortable, making it difficult to use in the daily home-testing environment.

### 2.2. Radar-Based Respiration Sensing

Doppler radar [12,13] can identify the heart and breathing rates of the human body by capturing wireless signals modulated by physiological motion phase. WiSpiro [24] uses continuous wave signals to accurately redirect radio waves to subjects’ chest to precisely monitor breathing from a distance whilst they sleep. Vital-Radio [16] uses FMCW radar technology to track human respiration and heartbeat by measuring the periodic changes in the time it takes for signals to be reflected from the chest back to the device. A series of pulses is transmitted to the target by using the impulse-radio UWB radar [14,15], and vital signs, such as respiration and heart rate, are extracted from the frequency spectrum of the received signals. These technologies provide respiration detection in high precision; however, they rely on specialised equipment and require some special customised hardware, and the system is complex and costly, which limits their deployment in daily family scenarios.

### 2.3. Wi-Fi-Based Respiration Detection Sensing

Halperin et al. [25] proposed the method to modify the firmware of the network card. Accordingly, CSI estimated by the network card can be directly obtained, which provides a foundation for the research of directly using commercial Wi-Fi to sense the environment. To our knowledge, Wi-Sleep [26] is the first sleep-monitoring system to use CSI collected by off-the-shelf Wi-Fi devices. Liu et al. [17] proposed the method of leveraging the CSI amplitude to estimate the breathing and heart rates of people during sleep. PhaseBeat [18] and TensorBeat [19] exploit phase difference between two antennas to eliminate the phase offset and detect the respiration rate. These methods experimentally demonstrate that CSI can detect respiration rate, but it does not provide theoretical foundation on the principle. Wang et al. [20] proposed a theoretical model of respiration sensing based on Wi-Fi signals in the Fresnel region and analysed and verified the factors influencing the detectability of the breath signals, which leads to the ineffective detection of the location of respiratory blind spots. On this basis, FullBreathe [21] takes advantage of the CSI amplitude and phase changes monotonously at partial breathing depth to estimate the respiration rate by combining the CSI amplitude and phase of the two antennas. We found that this improved method can solve the blind spot problem at some breathing depths; however, in other breathing depths, there may be a situation where neither the amplitude nor the phase of CSI can be effectively detected. Recently, FarSense [27] proposed that the ratio of CSI readings of two antennas be used as the respiration-sensing feature to expand the range of perception. ResBeat [28] uses bimodal CSI data to solve the problem of poor detection in a particular location. These methods improved the original methods and detection performance; however, they did not completely overcome the shortcomings of directly detecting respiratory signals using the CSI value.

## 3. Human Respiration Sensing 

In this chapter, we first introduce the basics of CSI. Then, we present the respiration-sensing model based on the Fresnel zone and analyse the limitations of CSI waveform in respiration sensing. Finally, we characterise the Doppler effect in respiratory movement and derive the respiration-sensing model with Doppler frequency shift to analyse the feasibility and specific method of extracting Doppler information from CSI.

### 3.1. CSI Primer

When the wireless signal propagates in space, certain phenomena, such as reflection, diffraction and scattering on the surface of the object, occur. The received signal is superposition of multiple paths called multipath effect. The signal undergoes complex multipath changes, which can be expressed in the form of the channel impulse response (CIR) in the time domain and the channel frequency response (CFR) in the frequency domain.

(1) $$h\left(t\right)=\sum_{n=1}^{N}a_{n}e^{-j\theta_{n}}\delta \left(\tau -\tau_{n}\right),$$

where, $a_{n}$, $\theta_{n}$ and $\tau_{n}$ are the amplitude attenuation, initial phase offset and time delay of the nth propagation path, respectively; and *N* is the total number of paths.

According to the physical layer definition of the IEEE 802.11n protocol [29] Wi-Fi signals are transmitted in the form of orthogonal frequency division multiplexing (OFDM). The OFDM channel of 20 MHz consists of 56 subcarriers (subchannels), each of which can be considered a narrowband channel. The researchers [25] modified the firmware of the Intel 5300 network interfere card to ensure that the CFR of the 30 subcarriers could be obtained from each transmitted packet and output as CSI.

### 3.2. Respiration Sensing in the Wi-Fi Fresnel Zone 

#### 3.2.1. Fresnel Zone

**Figure 1:**
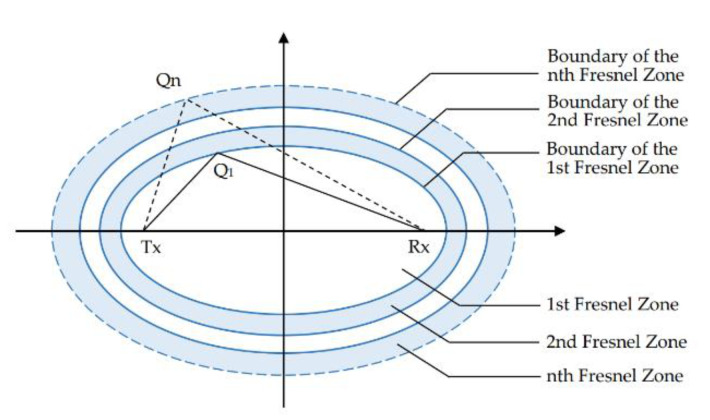
Fresnel zone.

The principle of Fresnel zone originated from the research of interference and diffraction of light. In the context of radio propagation, this concept refers to a series of concentric ellipse regions with the position of the transceiver as foci (Figure 1). The ellipse of a wavelength λ in the Fresnel zone satisfies the following formula:

(2) $$\left|T_{x}Q_{n}\right|+\left|R_{x}Q_{n}\right|-\left|T_{x}R_{x}\right|=n\lambda /2,$$

where $T_{x}$ represents the position of transmitter, $R_{x}$ represents the position of the receiver, and $Q_{n}$ represents any point on the nth ellipse boundary. The region within the innermost ellipse is defined as the first Fresnel zone. The region between the first and the second ellipses is defined as the second Fresnel zone. The region between the (n−1)th and the nth ellipse is defined as the nth Fresnel zone.

The length of the reflected path increases as objects move across the Fresnel zone away from the centre in proper order. According to the formula, every time after a Fresnel zone boundary, the reflection path length increases λ/2, and the phase increases π. The phase difference between the direct signal arriving through the LOS path and the signal reflected through the object to the receiver continuously increases. The superposition of the signal strength also alternately increases and decreases with the alternate change of the two signals in phase or out of phase.

#### 3.2.2. Respiration-Sensing Model

**Figure 2:**
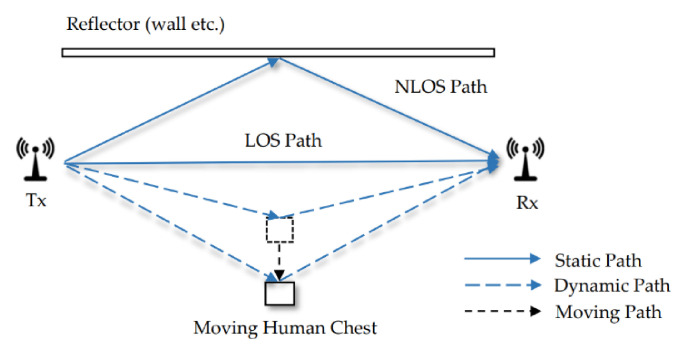
Indoor multipath environment with human movement.

Figure 2 shows the signal propagation through multiple paths in a typical indoor scenario. The image shows a moving human body and a reflector, such as walls in space. Part of the signals emitted by TX travels along the LOS path to RX, and part of travel along the NLOS path is reflected by an object. When only the person is moving, the path reflected by the body dynamically changes, whilst the other paths remain stationary.

According to the previous work [30], the multiple path signals reaching the receiver can be divided into static and dynamic components. The static component consists of the LOS path and the paths in reflection of stationary objects in space. The dynamic component is the sum of dynamic paths reflected from the moving objects. Therefore, the mathematical form of CSI can be expressed as follows:

(3) $$H\left(f,t\right)=H_{s}\left(f\right)+H_{d}\left(f,t\right)=\sum_{l=1}^{L}a_{l}\left(f\right)e^{-j2\pi d_{l}/\lambda }+\sum_{k=1}^{K}a_{k}\left(f,t\right)e^{-j2\pi d_{k}\left(t\right)/\lambda },$$

where $H_{s}\left(f\right)$ is the static component; $H_{d}\left(f,t\right)$ is the dynamic component of time t; L and K represent the number of static and dynamic paths; a is the attenuation; and d is the length of the reflection path.

The dynamic path discussed in this study refers to the path of changes caused by periodic breathing through the reflection of the thoracic cavity in the indoor environment. A breathing cycle consists of inflation and deflation of the lungs with pauses between each cycle. When inhaling facing the link, the lungs are inflated, and the chest is close to the transceiver; when out haling, the lungs are deflated, and the chest is away from the transceiver.

#### 3.2.3. Respiration Sensing by CSI Amplitude and Phase

From the principle of the Fresnel zone previously described, we know that the amplitude of CSI can produce a fragment of the sinusoidal-like waveform when an object moves a distance of λ between the Fresnel zones. With regard to breathing movement, the chest anteroposterior displacement is 4.2–5.4 mm, and the mediolateral dimension is 0.6–1.1 mm during normal exhalation and inhalation [31]. The length of the path reflected through the chest changes by approximately twice its displacement. When the centre frequency of the signal is 5.8 GHz (λ is approximately 5.17 cm), the phase change of dynamic component caused by chest displacement is in the range of 60°–150° in a breathing cycle. 

**Figure 3:**
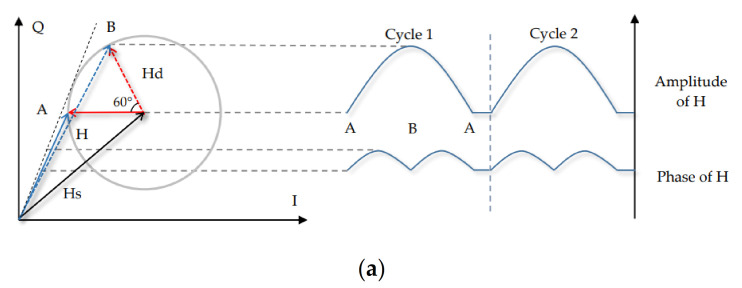

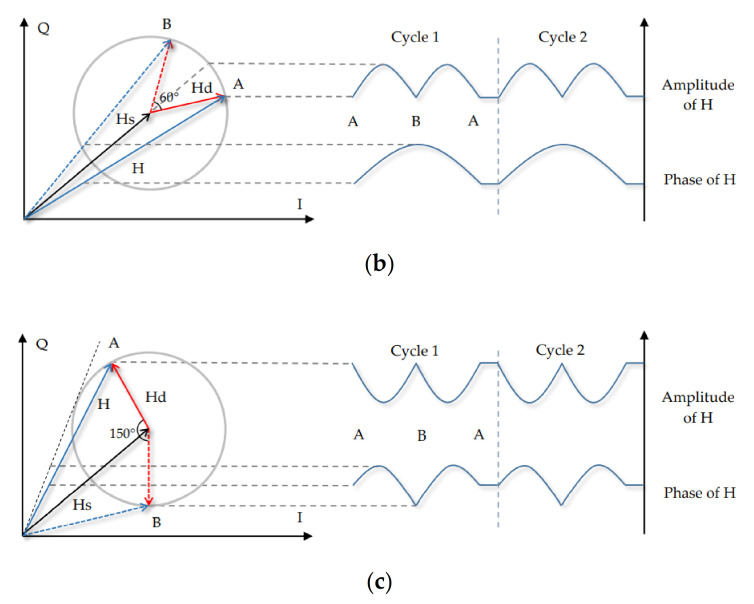
Variations of the resultant CSI amplitude and phase at different depths in various locations. (**a**) Case 1. (**b**) Case 2. (**c**) Case 3.

Therefore, the changes in the waveform caused by the respiratory signal do not constitute a complete waveform. Figure 3 simulates the variations in the amplitude and phase of the resultant CSI induced by breathing at different depths in various locations in the Fresnel zone. Figure 3a–c exhibit the phase change of 60° and 150° due to the breathing depth. Points A and B represent the nearest and furthest points from the chest to the link. The dynamic component rotates from A to B and again to A, representing one respiratory cycle. The static component maintains a fixed amplitude and phase during the chest displacement. The displacement range of the breath is in the millimetre scale; thus, the amplitude of the dynamic component can be considered to be basically fixed and the dynamic component mainly affected by changes in phase.

Case 1: Figure 3a presents the situation when moving near the middle of the Fresnel zone. During the rotation from A to B, the amplitude of H (the resultant CSI) monotonically increases, whilst the phase experiences the maximum value at the tangent line; accordingly, it increases and then declines. At this time, the CSI amplitude has a great extent of detectability.

Case 2: Figure 3b exhibits the situation near the boundary of the Fresnel zone. In contrast with Case 1, $H_{d}$ goes in phase with $H_{s}$, as long as the H experiences constructive interference; thus, the amplitude of H increases at the early stage and then decreases, whilst the phase kept monotonically increasing. At this time, the phase information of CSI can be used to better detect respiration.

Case 3: Figure 3c denotes the special case when the object is at the Fresnel zone boundary when the 150° phase change is caused by breathing. In the anticlockwise rotation interval from A to B, the amplitude and phase of CSI experience maximum values. In this case, large errors may occur regardless of the amplitude or phase used.

The above theoretical analysis indicates that the depth and location of breathing will affect the detection of signal. Monotonous amplitude or phase changes exist in the smallest-depth breathing; thus, an optimal detection location can be observed in the middle or boundary of the Fresnel zone. Non-monotonic amplitude and phase changes exist in the largest-depth breathing. At this time, the CSI value cannot effectively sense respiration. This notion indicates that weaker respiratory signals are easier to recognise; however, weaker signals are more susceptible to environmental noise and multipath effects.

In multi-carrier systems, such as OFDM, the Fresnel zone of each carrier has overlapping boundaries, and the difference between the corresponding boundaries will become larger with the increase in the number of zones. This situation may be good for one subcarrier but bad for another. In this case, finding the best perceptual location in the Fresnel zone can be even more complicated.

### 3.3. Respiration Sensing Based on Doppler Effect 

#### 3.3.1. Respiration-Sensing Model Based on Doppler Effect

In view of the limitations of detecting breathing signals with CSI waveform, we try to extract the part that directly reflects the respiratory movement from CSI for detection. The Doppler effect generated by thoracic movement during breathing inspired us to extract Doppler frequency from CSI for respiration sensing.

The Doppler effect refers to the phenomenon that the frequency of received signal changes when the receiver of the signal moves relative to the transmitter. In the environment of wireless sensing, the transmitted signal is reflected from the moving object to the receiver when the receiver and transmitter remain static, and the carrier frequency of the reflected signal has a Doppler frequency shift [32]. This concept can be expressed as follows:

(4) $$f_{doppler}=2f\frac{v}{c},$$

where v is the speed at which the object is moving, f is the original frequency at which the signal is sent and c is the speed of light.

The fundamental reason for Doppler shift is the change in path length of signal propagation. At time t, the delay of the kth path can be expressed as $\tau_{k}\left(t\right)=\tau_{k}\left(0\right)+\Delta d_{k}\left(t\right)/c$, The CSI in Equation (3) can also be expressed as a superposition of dynamic paths modulated by the Doppler shift:

(5) $$H\left(f,t\right)=H_{s}\left(f\right)+\overset{K}{\underset{k=1}{\sum }}\vec{a_{k}}\left(f,t\right)e^{j2\pi \int_{0}^{t}f_{d_{k}}\left(u\right)du},$$

where $f_{d_{k}}$ is the Doppler shift, and $\vec{a_{k}}\left(f,t\right)$ is the attenuation and initial phase offset for the kth path. The complex channel gain with Doppler shift modelled by the multipath in [33] has the similar expression to (5). The above equation demonstrates that phase change caused by Doppler shift exists in CSI, and it only exists in each dynamic path. However, the phase caused by the Doppler phase is mixed with the phase of time delay, which cannot be obtained by directly analysing the phase. In addition, the chest displacement is small, and the change speed is slow, resulting in a small Doppler shift. In the complex multipath indoor environment where the amplitude of the static component is much larger than that of the dynamic component, the target frequency is easily submerged in the static clutter and noise. Therefore, the Doppler frequency component is difficult to directly extract from CSI.

#### 3.3.2. Extraction of Doppler Information from CSI

According to the previous definition, CSI is the frequency domain superposition of the CIR. The CIR signal does not involve complex time-varying components; however, it intuitively represents the attenuation and phase offset induced by time delay of the signal in different paths at the current moment. The collected CSI contains 2D time. The time *t* of arrival of each packet is the fast time dimension, and the propagation time delay *τ* of the multipath signal is the slow time dimension. The CIR is obtained by time–frequency transformation of CSI in the slow time dimension, whilst the Doppler phase term is only related to the fast time dimension; hence, the Doppler component remains unchanged during this process. Therefore, the process of conversion to CIR can retain the Doppler phase term and eliminate the phase interference caused by the delay.

Therefore, we perform multipath decomposition to CSI. Fine-grained path information can be obtained by separating multipath signals in the time domain, and the dynamic path containing Doppler shift can be resolved. Specifically, the CIR is obtained by taking the IFFT of each CSI along the slow time dimension:

(6) $$h\left(\tau ,\;t\right)=h_{s}\left(\tau \right)+h_{d}\left(\tau ,t\right)=\sum_{l=1}^{L}a_{l}\delta \left(\tau -\tau_{l}\right)+\sum_{k=1}^{K}a_{k}\left(t\right)e^{-j2\pi \int_{0}^{t}f_{d_{k}}\left(u\right)du}\delta \left(\tau -\tau_{k}\left(t\right)\right),$$

where $h_{s}\left(\tau \right)$ and $h_{d}\left(\tau ,t\right)$ are the time domain responses of the static and dynamic components, respectively.

The CSI has 30 subcarriers with a subcarrier interval of 312.5 kHz. Accordingly, the corresponding $h\left(\tau ,\;t\right)$ contains 30 delay units (path units) with a time interval of 50 ns, and each fixed unit includes the sum of multipath signals in the current delay interval. Considering that the breathing movement is weak, the breathing signal is difficult to extract from the dynamic signal after multiple reflections. Therefore, under the experimental environment in this study, the dynamic component is considered to exist in the first three delay units. The CIR values of all 30 units are simplified by a matrix:

(7) $$h\left(t\right)=\left[a_{1}+a_{1}^{\prime }e^{-j2\pi f_{d_{1}}t},\;a_{2}+a_{2}^{\prime }e^{-j2\pi f_{d_{2}}t},a_{3}+a_{3}^{\prime }e^{-j2\pi f_{d_{3}}t},a_{4},\ldots ,a_{30}\right],$$

where $a_{1}$ represents the amplitude of the sum of static signals in the first unit, and $a_{1}^{\prime }$ represents the amplitude of dynamic signals in the first unit.

The comparison of Equations (5)–(7) illustrates that the multipath decomposition separates the path elements containing the Doppler shift, which reduces the interference of static clutter. In the first three path units, the phase of the dynamic path is mainly determined by the Doppler shift, which creates the conditions for extracting the Doppler shift from the dynamic path. As an example, Fourier transform is applied to the first path unit along the fast time dimension, and the following expression is obtained:

(8) $$H\left(f\right)=F\left[a_{1}\;]\delta \left(f\right)+F[a_{1}^{\prime }\right]\delta \left(f-f_{d}\right),$$

Therefore, Doppler information can be extracted from CSI. According to the previous analysis, the static component can be considered a constant, and it is much larger than the dynamic component. The value of $f_{d}$ is close to the zero frequency; thus, the zero-frequency component may overwrite the Doppler frequency component. In this case, we extracted the respiratory signal from the accumulated Doppler spectral energy after removing the static clutter. See Section 4.4 for the detailed steps and results.

## 4. Reparation Rate-Monitoring System

### 4.1. System Overview

**Figure 4:**
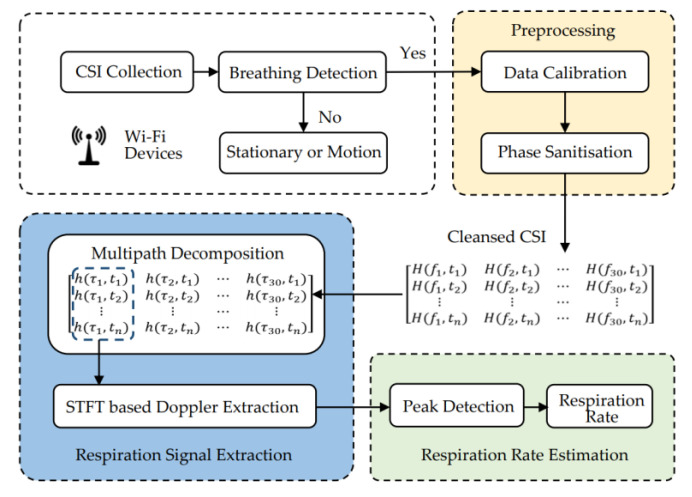
System architecture.

We designed a system to monitor human respiration by using CSI data collected from commercial Wi-Fi devices. The specific process is shown in Figure 4, which mainly includes four modules: data extraction, preprocessing, respiration signal extraction and respiration rate estimation.

In the data extraction module, the original CSI data are collected by commercial Wi-Fi devices. The fluctuation in CSI is observed to determine the existence of humans in the current environment, and respiration can be detected. In the data preprocessing module, we first remove high-frequency noise and unstable part of the static component in the data. Then, we process the linear fitting error of the unsynchronised phase information to obtain usable phase information. Finally, we obtained cleansed complex CSI information. In the respiration signal extraction module, we first perform multipath decomposition. The IFFT of CSI along the fast time dimension at each moment is used to obtain the CIR. Then, we extract the Doppler information in the first three multipath units containing dynamic components along the fast time dimension. STFT is applied to each multipath unit to obtain the spectrum of the Doppler shift. Finally, we remove the static component of the spectral energy and extract the respiratory signal from the zero-frequency accumulated Doppler spectral energy waveform. In the respiration rate estimation module, the respiration rate was calculated by the method of peak-to-peak distance detection.

### 4.2. Breathing Detection

Our system estimates human breathing rate at rest; hence, we need to determine if the environment is suitable for breathing detection. CSI can be affected by human movement in the environment, and the value of CSI irregularly fluctuates when someone moves around, stands up or sits down in space. The fluctuation at this time is more violent compared to the static state or no one state.

Accordingly, a statistical feature-based method was adopted to detect respiratory signals and calculate the sum of the standard deviations of the CSI values within the sliding time window of all subcarriers. Then, a smooth statistic curve was obtained with a moving average filter. In our experimental scenario, we assumed that no one was present in the initial environment. After someone approached the device, sat down and remained stationary, we calculated the respiration rate. The statistics start small and then go back to a smaller value after a sharp rise and fall. We set the threshold to 0.1. When the statistics is above the threshold, we assume someone is nearby. When the statistics is below the threshold for the first time, we collect the CSI for the subsequent operations.

**Figure 5:**
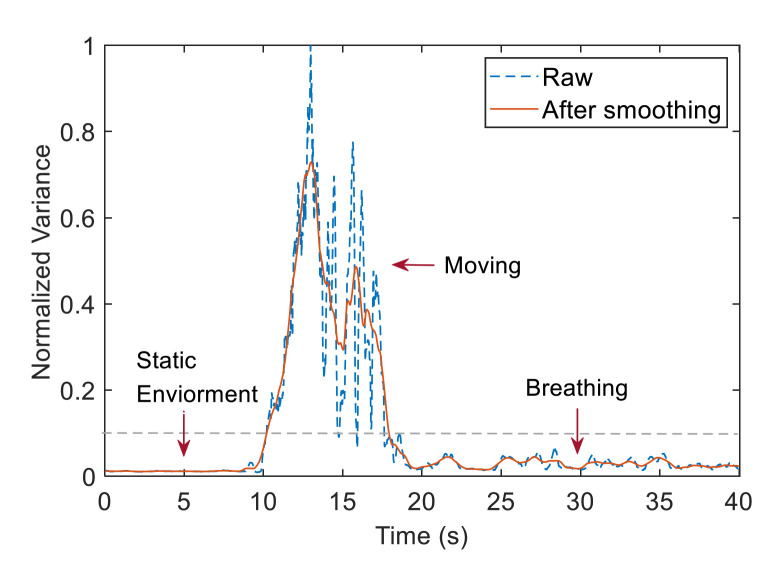
Breathing detection.

Figure 5 exhibits the variation of the sliding standard deviation and smoothing characteristics during the whole process. The data remain stable when no one can be seen within the range. Meanwhile, the data wildly fluctuate when someone comes up and sits down. After the person is still and breathing normally, the standard deviation data return to a more stable state with slight fluctuations. Therefore, this detection algorithm can eliminate the static environment when no one is present and effectively identify the static situation when people are in.

### 4.3. Preprocessing

#### 4.3.1. Data Calibration

The change of the internal state of the device and the electromagnetic interference in the environment often introduce high frequency noise in the CSI. These noises may interfere with the extraction of weak respiratory signal or even drown the signal. Thus, the raw data received must be preprocessed.

First, the previous analysis indicated that the static and dynamic component amplitudes are basically stable during breathing; accordingly, the CSI periodically fluctuates on the horizontal line. We use Hampel Filter to detrend the unstable part in the static component. Specifically, we set the sliding window of the filter to 400 samples with a threshold of 0.05. After the basic trend of the original data is obtained, we determined the difference between the basic trend and the trend at the initial moment. Then, we remove this part of the trend from the original data. The main static components in this method are retained to avoid the removal of the unseparated dynamic components to remove the main static components in the following steps.

**Figure 6:**
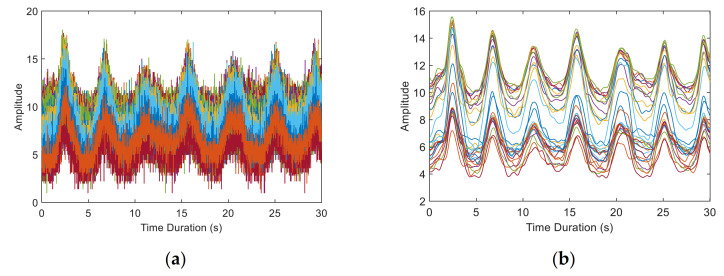
CSI amplitude of 30 subcarriers before and after data calibration. (**a**) Raw CSI amplitude. (**b**) CSI amplitude after calibration.

Then, we use Savitzky–Golay Filter [34] to filter out the high frequency noise. This filter can retain the detailed features of the signal better whilst filtering smoothly and reduce the distortion compared with the traditional filter. Figure 6 exhibits CSI amplitude of 30 subcarriers before and after data calibration. The high frequency noise is effectively eliminated, and the changes of each subcarrier can be clearly seen.

#### 4.3.2. Phase Sanitisation

Certain problems, such as the clock of the receiver and the transmitter is not synchronised, and the hardware is not fully compensated, occur due to the circuit characteristics of the equipment. Thus, the original phase of the measured CSI presents a tendency of random distribution and cannot be directly used for detection. We design a special phase sanitisation step to correct the error between the measured and the real values. 

According to previous studies [35,36], the main phase errors introduced are as follows:

-  Carrier frequency offset (CFO): The carrier frequency offset is caused by the incomplete synchronisation of the carrier frequency generated by the oscillator of the receiver and transmitter. The CFO corrector compensated the carrier frequency; however, a residual CFO was still present, which is noted as $f_{c}$. In the IEEE802.11n standard, the frequency is allowed to be as high as 100 kHz [29]. Accordingly, a large phase uncertainty is introduced. The CFO is determined only by the hardware characteristics; hence, it is a constant that does not change over time.
-  Sampling frequency offset (SFO): In the sampling process of analogue-to-digital convertor, the sampling frequency is offset due to the unsynchronised clock. This phenomenon introduces a time offset termed as $\tau_{s}$ between the adjacent sampling points and causes a phase rotation error in the subcarrier. SFO can be considered stable for short periods of time.
-  Packet detection delay (PDD): The packets of the Wi-Fi signals are transmitted in the frame format specified by the protocol. The packet detector detects the arrival of a packet by using the preamble. This situation introduces the packet detection time delay $\tau_{p}$, resulting in a phase rotation error. PDD is random for packets arriving at different times.

The above error sources demonstrate that the measured phase of the subcarrier can be expressed as follows:

(9) $$\tilde{\varphi_{k}}=\varphi_{k}-2\pi \Delta f\left(\tau_{p}+\tau_{s}\right)k+2\pi f_{c}+Ζ,$$

where $\varphi_{k}$ is true phase that we attempt to acquire, k is the subcarrier index specified by IEEE802.11n, Δf is the spacing between two adjacent subcarriers (312.5 kHz), and Ζ is Gaussian additive white noise.

We cannot calculate the error of each packet with only NIC. However, all significant phase errors in CSI are linear to the subcarrier index; hence, the phase error term can be regarded as a linear function of subcarrier number k. We used a method similar to SpotFi [37] to search for linear fitting. The key idea is to find out the linear phase errors of the original phase and remove them. We use the minimum mean square error method to calculate the linear function closest to the original phase. Slope *α* and intercept *β* of the fitting linear function are as follows:

(10) $$\left(\alpha ,\beta \right)=arg\;\underset{\alpha ,\beta }{\min}\sum_{k=1}^{K}{\left(\tilde{\varphi_{k}}+\alpha k+\beta \right)}^{2},$$

In particular, Equation (3) illustrates that the CSI phase of each path also has the form of a linear function. The total $H\left(f,t\right)$ phase no longer linearly varies with the subcarrier index like a single path. Therefore, the phase error can be eliminated by extracting the linear part. Finally, the cleansed phase can be obtained by removing the linear fitting part. The phase after sanitisation is as follows:

(11) $$\varphi_{k}=\tilde{\varphi_{k}}-\alpha k-\beta ,$$

**Figure 7:**
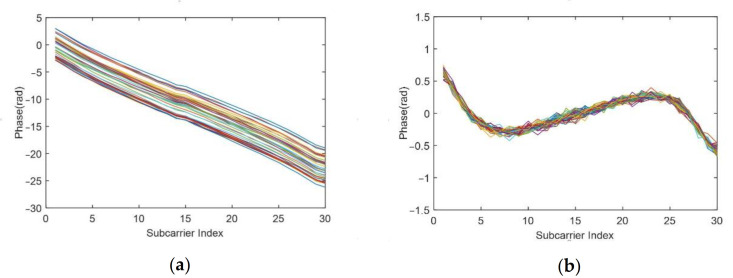
CSI phase of 40 packets before and after sanitisation. (**a**) Unwrapped CSI phase. (**b**) CSI phase after sanitisation.

Figure 7a shows the unwrapped original CSI phase. The phase distribution presents a linear trend. However, we find that the unwrapped phase presents a two-segment splicing around subcarrier 15. This situation occurs because the 30 subcarriers outputted by the NIC are selected from the 56 subcarriers actually calculated, and the extracted subcarrier index is [−28, −26, −24, … −2, −1, 1, 3, … 25, 27, 28]. We interpolate 30 subcarriers into 56 subcarriers according to the subcarrier index for accurate fitting. After the processed phase is obtained, the 30 subcarriers are extracted from it. Figure 7b presents the phase after sanitisation. The phase after processing is stable in a small frequency range and does not linearly change with the subcarriers, which is used as a reference for the real phase. In combination with the preprocessed amplitude, we can obtain usable complex CSI.

### 4.4. Respiration Signal Extraction

The principle of using Doppler information to sense respiration is that the chest displacement produces the Doppler shift during the inhalation and exhalation. Thereafter, the chest stays still for a short time without a Doppler shift. This notion means that the Doppler change period corresponds to the breathing period. 

We perform multipath decomposition for the preprocessed CSI to separate the dynamic path unit containing Doppler information. Specifically, 30 point IFFT is applied to each packet to obtain the CIR at each moment. The data from the first three path units that include Doppler information are extracted. Then, we use the time–frequency analysis tool STFT to obtain the Doppler shift spectrum. Calculating specific frequency values in the STFT requires a long integration time to ensure the desired frequency resolution. However, this integration time may exceed the time that the Doppler frequency exists in each period. The Doppler spectral energy changes also remain periodic because the Doppler shift periodically occurs. When the time window of STFT slides from the rest moment between breathing cycles to the breathing moment in the next cycle, the accumulated Doppler energy gradually increases; otherwise, the accumulated energy decreases from the breathing moment to the rest moment. Thus, a breathing cycle produces a peak in the spectral energy waveform.

Specifically, STFT was applied to the dynamic path unit. The window length was set as 1 s, and the step size was 0.1 s. The Doppler frequency component falls within the zero-frequency bin, in which a DC component also exists. Considering that the spectral energy of the DC component basically does not change with time, we subtracted the spectral minimum energy to obtain a clearer energy waveform.

**Figure 8:**
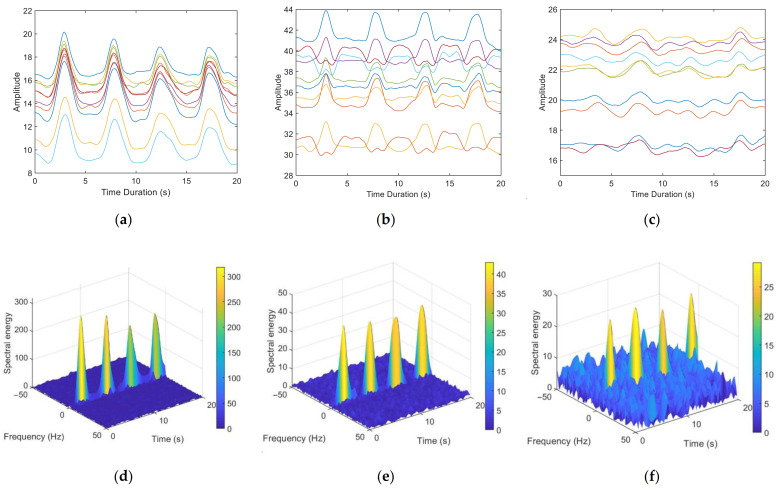
CSI waveforms and corresponding 3D spectrogram of respiration signal extraction results of the three breathing patterns: (**a**,**d**) deep breathing, (**b**,**e**) normal breathing and (**c**,**f**) weak breathing.

We chose to test our method in a scenario where multipath effects are severe. Figure 5 illustrates the CSI waveform and the corresponding spectrogram for three breathing patterns: deep breathing, normal breathing and weak breathing. Figure 8a–c shows amplitude waveforms of the first ten subcarriers with the largest variance of CSI obtained by the method in [17]. Figure 8d–f are the results of the corresponding 3D spectrogram. In the case of deep breathing, the four breaths within the sampling time can be clearly identified from the CSI amplitude waveform (Figure 8a) and the zero-frequency energy waveform in the spectrum (Figure 8d). In the case of normal breathing, most subcarriers in Figure 8b clearly show the corresponding peaks of the four breaths. However, the remaining subcarriers show a trend opposite to the change of respiratory signals, with multiple peaks appearing within a breathing cycle, which is consistent with the theoretical analysis in Section 3.2.3. By contrast, four breaths can be clearly identified in Figure 8e. In the case of weak breathing, the respiratory signal is interfered by clutter. Figure 8c demonstrates that respiration is difficult to identify by CSI amplitude. Nevertheless, we can still clearly see the four peaks corresponding to the respiratory signals in Figure 8f.

### 4.5. Respiration Rate Estimation

We extracted the spectral energy waveform at the zero frequency of the spectrogram in Figure 8 to identify the respiration rate. We have three energy waveforms in the path units to choose from. In most cases, the waveform extracted from the first path has the similar periodicity as the respiratory signal and can be used for rate estimation. To better estimate the respiration rate, we leverage the method based on recurrence plot in the reference [26,38] to select the waveform with the best periodicity.

Each peak of the respiratory signal corresponds to a respiratory cycle. We exploit the peak identification algorithm to estimate the respiration rate, which has higher time resolution than the FFT method. Specifically, fake peak removal algorithm [17] is used to find the peak location for each breathing cycle and calculate the intervals between adjacent peaks. Taking the average peak-to-peak interval as the breathing cycle C, the respiration rate estimation result is 60/C bpm (breath per minute). 

## 5. Evaluation

### 5.1. Experiment Configuration

**Figure 9:**
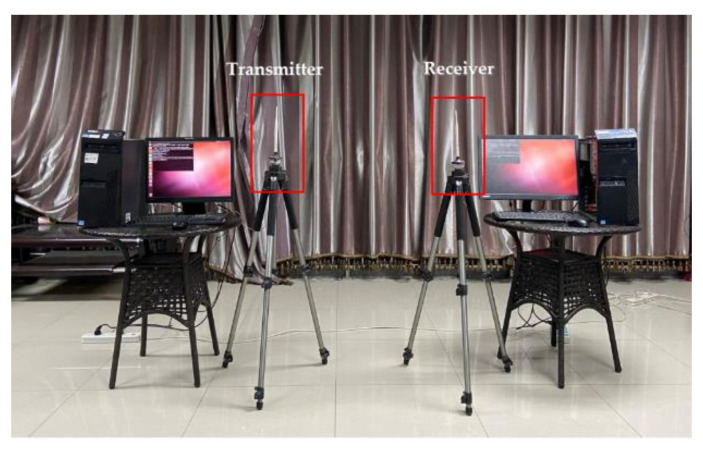
Experiment devices.

We implemented our system in two commercial computers (i.e., Lenovo M8500t) with Intel 5300 NIC. The operating system is Ubuntu 14.04 LTS. The two computers, which act as a sender and a receiver, are equipped with one antenna respectively. The device is shown in Figure 9. According to the frame format of data transmission specified by IEEE 802.11n, the Linux 802.11n CSI Tool [39] which modified the network card firmware, was used to collect Wi-Fi CSI data, and the device worked in the monitor mode. In terms of data transmission, we set the working frequency band as #64(5.32 GHz) and the signal bandwidth as 20 MHz to reduce electromagnetic interference in space. A transmission rate of 100 packets per second is adopted. Each packet can extract a CSI matrix of the size of 1 × 3 × 30, representing the CSI complex value of the subcarriers received by each of the three antennas on a network card.

**Figure 10:**
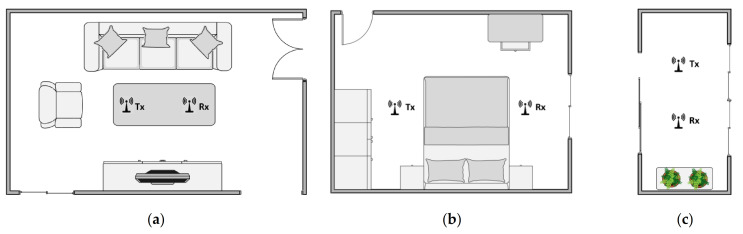
Plan of the experimental scenarios. (**a**) Living room. (**b**) Bedroom. (**c**) Balcony.

To evaluate the performance of our system in the actual scenario, the transmitter and receiver are placed in the indoor scenario, and the experimenter sits on the side of the link and breathed on the link. The application scenario of our system is daily home health monitoring. Thus, three typical indoor environments are selected to evaluate the performance of our system: a 5.8 m × 3.4 m living room, a 3.6 m × 2.7 m bedroom and a 2.9 m × 1.3 m balcony. The plan of the experimental scenarios is shown in Figure 10. The normal respiratory rate of adults is 12–20 bpm [40]. The experimenter was allowed to normally breath for a specified period of time rather than consciously controlling his or her own breathing according to a set breathing program to obtain the measurement results of the device under the most realistic conditions. The respiration rate recorded by the NEULOG Monitor Logger Sensor [41] is employed as the ground truth.

### 5.2. Performance of Respiration Rate Estimation

The purpose of our system is to obtain the respiration rate of the human body. Thus, the estimation error between the respiration rate calculated by the algorithm in this study and the ground truth is chosen as the metric to measure the accuracy. Estimation error statistics are the common evaluation parameters in the field of respiration rate detection.

**Figure 11:**
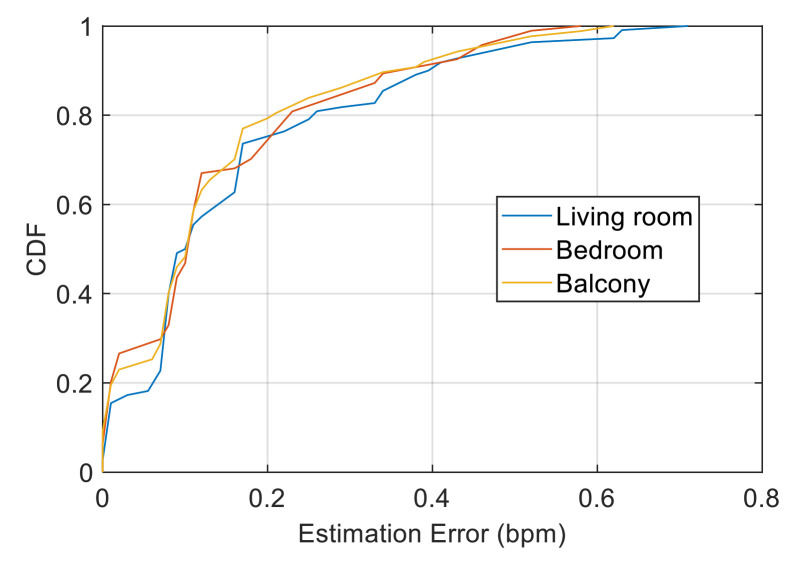
Performance in the living room, bedroom and balcony scenarios.

We first evaluated the performance of our system under different scenarios. Figure 11 presents the cumulative distribution function (CDF) of the respiration rate estimation errors in the living room, bedroom and balcony scenarios. The maximum estimated error was less than 0.7 bpm, and the median errors for the three scenarios were 0.10, 0.11 and 0.11 bpm. Our system achieved high estimation accuracy in different indoor scenarios. Over 90% of the estimated errors are less than 0.4 bpm, of which the proportion of the balcony scenario is slightly higher than the other two scenarios. However, the error distribution of the three scenarios is overall similar. Theoretically, a severe multipath effect can be observed in the narrow balcony compared with that in the living room, and the path reflected through the chest will be increased, making the respiratory signal slightly stronger. However, static clutter is more intense and has stronger interference with respiratory signals. Accordingly, the performance decreases. However, no such trend can be seen from our experimental results, indicating that the Doppler information extracted by our system has excellent anti-multipath ability and can be basically unaffected by the scenario, which effectively reflects the breathing movement.

We selected two schemes in previous work as the benchmark to compare the performance: the method based on CSI amplitude [17] and the method based on phase information [18]. In the amplitude-based method, multiple subcarriers with large variance of CSI amplitude were selected for peak detection, and the weighted average of the results was used to determine the final respiration rate. In the phase-based method, the CSI of the two antennas is used to calculate the phase difference. One of the subcarriers with higher sensitivity to respiratory signals was selected. The approximate coefficient obtained after four-level discrete wavelet transform was used to monitor the respiration rate.

**Figure 12:**
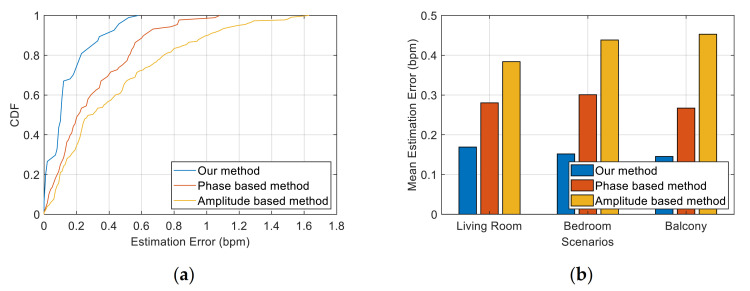
Performance of our method and two benchmark methods (amplitude-based and phase-based). (**a**) CDF of the estimation error of the three methods in the bedroom scenario. (**b**) Mean estimation error of the three methods in the living room, bedroom and balcony scenarios.

Figure 12 exhibits a comparison of the estimation errors between our method and the two benchmark methods. Figure 12a shows the CDF in the bedroom scenario. The maximum errors of the two benchmark methods and our method are 0.58, 1.08 and 1.63 bpm, and the median errors are 0.10, 0.21 and 0.31 bpm, respectively. In our method, 97% of the test data had an error of less than 0.5 bpm. However, less than 76% of the test data in the benchmark method had an error of less than 0.5 bpm. This finding indicates that the performance of our system is superior to the traditional scheme and achieves a high accuracy. Figure 12b compares the mean estimation error of respiration rate in different scenarios between our method and the benchmark method. The mean error of our method is stable at approximately 0.15 bpm, and the accuracy of the respiration rate calculated by our method is significantly higher than that of other traditional schemes. The reason is that our method only extracts the respiratory signal from the path that contains the target’s movement; thus, the extracted information is more accurate. This notion represents that our respiration signal extraction scheme based on Doppler information has the ability to solve the problem of the detectability of breath depth and position and maintain robustness under different scenarios.

### 5.3. Verifying the Detectability of Breathing Locations

In the previous section, we analysed the influence of the breathing position on the detectability of CSI the amplitude and phase. In this section, we evaluate the effect of breathing location on the performance of our method and the traditional method.

According to previous studies [42], more than 70% of the energy of the RF signal is transmitted through the first Fresnel zone, and the first 8–12 Fresnel zones are important for RF transmission. As previously mentioned in Section 3.2.2, each subcarrier in a multicarrier system has its own Fresnel zone; thus, it is difficult to know the overall situation after they overlap. We calculate the Fresnel zone of a subcarrier with a central frequency of 5.32 GHz. When the distance between the transmitter and receiver is 2 m, the first third Fresnel radii are 0.168, 0.237 and 0.291 m, and the eighth and twelfth Fresnel radii are 0.475 and 0.582 m, respectively. Therefore, we compare the estimation error between our method and the two benchmark methods in the range of 0.3 m to 0.8 m from the device. Specifically, the experimenter sits in the initial position, which is 0.3 m away at perpendicular bisector of LOS and moved backward 0.1 m in turn. The distance of each movement is greater than the maximum Fresnel zone interval, and the distance between adjacent zones continues to decrease. Therefore, the detectability is different for each location. A good system should not be affected by location detectability and can achieve good detection results in different locations.

Figure 12 depicts the mean estimation error of the respiration rate between our method and the two benchmark methods at different distances between the experimenter and the devices. We observe that our method has a high accuracy in these positions, and the estimation error is stable within the range of 0.10 bpm to 0.15 bpm. However, the estimation errors of the two benchmark schemes dramatically fluctuate. The estimation error of the amplitude-based method is within the range of 0.21 bpm to 0.42 bpm, whilst the phase-based method was within the range of 0.14 bpm to 0.39 bpm. The estimation error of the two benchmark schemes shows the opposite trend in the whole. For example, the phase-based method has the lowest estimation error at distances of 0.6 and 0.8 m, whereas the amplitude-based method has relatively high estimation error at these locations. 

**Figure 13:**
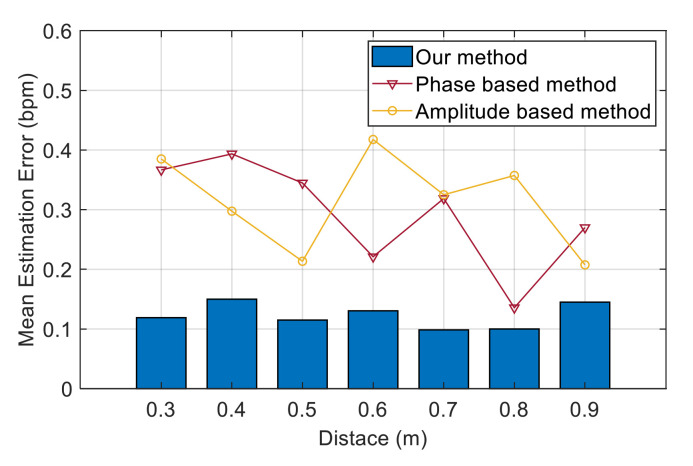
Performance of different distances between the experimenter and the devices of three schemes.

In the results of Figure 13, we can observe several situations: the CSI amplitude is more detectable than the phase (distances of 0.4 and 0.5 m); the CSI phase is more detectable than the amplitude (distances of 0.6 and 0.8 m); the amplitude and phase can achieve good detection results (distance of 0.9 m); and neither the amplitude nor the phase can obtain good detection results (distance of 0.3 m). This finding confirms the limitations we mentioned earlier of using CSI values to sense respiratory signals. Therefore, these experimental results indicate that our method can effectively solve the problem of position affecting the detectability of respiratory signals and achieve a stable performance of high-precision identification.

### 5.4. Impact of Various Factors

In this section, we analyse the major factors influencing respiration detection and evaluate their impact on our system. 

#### 5.4.1. Impact of Distance between the Transmitter and the Receiver

**Figure 14:**
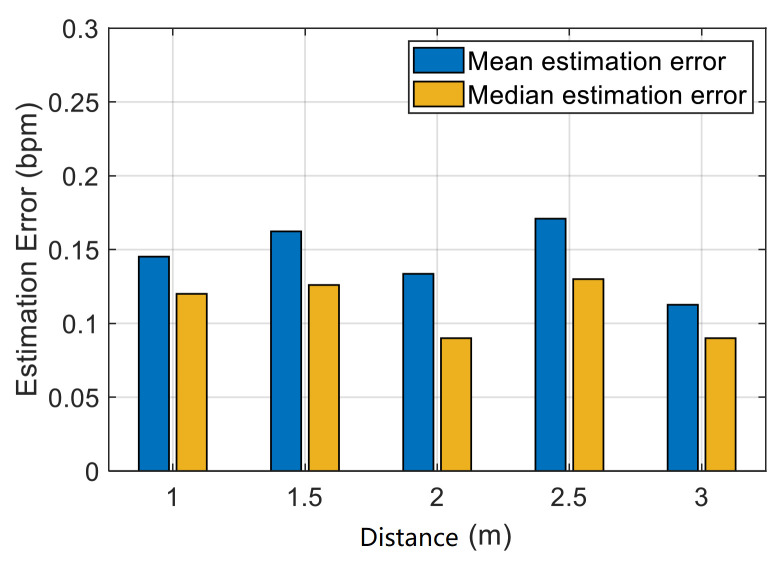
Impact of distance between the transmitter and the receiver.

In the actual scenario, Wi-Fi devices may be placed in any place; hence, the influence of different distances between the devices on respiration rate detection is considered. Figure 14 shows that the system can achieve high accuracy at distances ranging from 1 m to 3 m. A minimum mean estimation error of 0.13 bpm can be observed in the distance of 2 m. The maximum mean error in the distance of 2.5 m is 0.17 bpm. The closer the distance between the devices, the stronger the interference of direct waves will be. Meanwhile, the farther the distance, the weaker the received signal. Our experimental results suggest that the detection accuracy of the respiration rate does not significantly decrease with the increase in distance within the practical distance considered in our experiment, which indicates that our system has strong robustness.

#### 5.4.2. Impact of Sampling Rate

**Figure 15:**
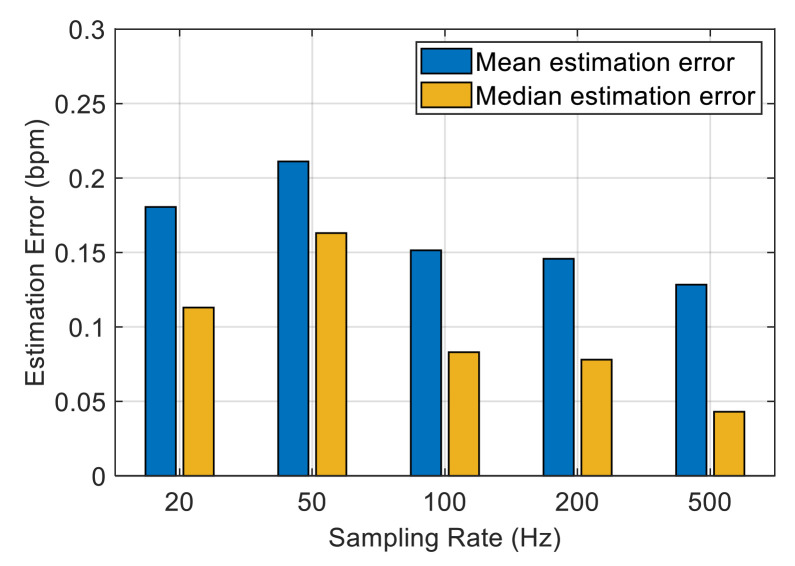
Impact of sampling rate.

We are interested in how the sampling rate affects the performance of the system. A higher transmission rate means more CSI values, which reflect the trend of respiratory signal in more detail, but it also increases the computational complexity of the system. Figure 15 presents the mean and median estimation errors of the data collected at different sampling rates in the bedroom scenario. The overall estimation error decreases with the increase in the sampling rate. When the sampling frequency exceeds 100 Hz, the average estimation error drops below 0.15 bpm. In our system, we use STFT to process the data, and a higher sample rate means more FFT points and a longer processing time. Therefore, we choose 100 Hz as the sampling rate of the experiment in this study.

## 6. Conclusions

In this study, we present a respiration rate-monitoring system that is applied in daily home health-monitoring scenarios by commercial Wi-Fi devices. This system uses Doppler information for breathing sensing.

From existing respiration-sensing model based on the Fresnel zone, we investigate that breathing depth and location affect the detectability of the breathing signal. Therefore, the method based on CSI amplitude and phase cannot achieve full state detection. We proposed a respiration-sensing model based on Doppler frequency using the principle of periodic Doppler shift produced by periodic thoracic movement during breathing to solve this problem. Moreover, we theoretically analysed the possibility of extracting Doppler information from CSI.

Weak Doppler generated by respiratory movement is easily submerged in static clutter caused by multipath effect in a real indoor environment. Therefore, we propose a method to extract Doppler information from CSI. First, the random phase shift is eliminated to obtain vector CSI. Then, IFFT is used for decompose multipath, and the signal in the dynamic path unit containing the Doppler frequency is extracted. Finally, the Doppler spectral energy is accumulated by STFT to calculate the accurate respiration rate.

We designed and implemented a complete respiration rate-monitoring system, including environmental detection, amplitude and phase preprocessing, multipath decomposition, Doppler spectral energy extraction and respiration rate estimation steps. We implemented our system with commercial Wi-Fi devices in three classic indoor home scenarios and evaluated its performance through extensive experiments with detection results of high precision in all scenarios. The results demonstrate good anti-multipath ability, robustness and ability to solve problems with the detectability of respiratory signals compared with traditional methods in different scenarios and locations. We provide a non-invasive, cost-effective, reliable respiration rate-monitoring program scheme for home health monitoring.
